# Unavoidable exposure to secondhand smoke in indoor places: a cross-sectional comparison to the Health Japan 21 (second term) objectives, 2022

**DOI:** 10.1265/ehpm.23-00055

**Published:** 2023-07-12

**Authors:** Satomi Odani, Takahiro Tabuchi

**Affiliations:** 1 Cancer Control Center, Osaka International Cancer Institute, 3-1-69 Otemae Chuo-ku, Osaka, Japan; 2 Graduate School of Medicine, Osaka University, 2-2 Yamadaoka, Suita, Japan

**Keywords:** Health Japan 21, MPOWER, Secondhand smoking, Smoke-free environment, Internet survey, Tobacco control

## Abstract

**Background:**

The second term Health Japan 21 aims at eliminating unwanted exposure to secondhand smoke (SHS) in society; however, the ambiguity of the term “unwanted exposure” complicates the evaluation of the program. In this study, we examined SHS exposure that occurred despite the efforts to avoid it (i.e. unavoidable SHS exposure) as a proxy for “unwanted SHS exposure”.

**Methods:**

Individuals aged 16–74 responded to a nationwide, Internet-based, self-reported survey. Frequency (daily/≥monthly) of SHS exposure in the past month was assessed for specific places (home/car/restaurant/cafe/bar/workplace/school/pachinko parlor) and any place. Unavoidable SHS was identified when respondents always tried to avoid but were exposed to SHS. The observed prevalence was compared to the target of Health Japan 21 (“Eliminate unwanted SHS exposure”, =0%). Analyses were weighted to account for the selectivity of the Internet-based sample.

**Results:**

Among overall (N = 25,672), those who always tried to avoid SHS (N = 14,971), and never smokers of combustible tobacco who always tried to avoid SHS (N = 10,416), the prevalence of daily SHS exposure was 12.4%, 5.7%, and 4.2%; ≥monthly SHS exposure was 34.0%, 21.4%, and 17.5%, respectively. Among never smokers, the adjusted prevalence ratio (APR) of daily unavoidable SHS exposure was significantly high in adolescents (age 16–19) (APR = 4.97, vs. age 60–74), less-educated individuals (APR = 2.37, vs. ≥some college education), and heated tobacco product (HTP) users (APR = 8.27, vs. nonusers). Among never smokers, daily unavoidable SHS exposure was highest in the home (3.4%), workplaces (2.3%), and pachinko parlors (1.3%); ≥monthly unavoidable SHS exposure was highest in workplaces (11.4%), restaurants/cafes/bars (10.0%), and the home (7.6%).

**Conclusions:**

Daily unavoidable SHS exposure was disproportionately high among adolescents, less-educated individuals, and HTP users. The prevalence of unavoidable SHS exposure did not reach the national target in any of the assessed indoor places; home and workplace were the dominant sources of unavoidable SHS exposure. The lack of comprehensive smoke-free laws provides inadequate protection against SHS that cannot be complemented by individual efforts. The authorities must ensure smoke-free environments for all.

**Supplementary information:**

The online version contains supplementary material available at https://doi.org/10.1265/ehpm.23-00055.

## Introduction

Secondhand smoke (SHS) is one of the most preventable cause of morbidity and mortality known to cause stroke, lung cancer, and coronary heart disease in adults [1–3]; children exposed to SHS are at increased risk for middle ear disease, respiratory symptoms, impaired lung function, lower respiratory illness, and sudden infant death syndrome [1–3]. Since there is no safe level of exposure to SHS, the only effective way to protect people is to provide a 100% smoke-free environment as called for by the World Health Organization Framework Convention on Tobacco Control (WHO FCTC) [4]. Partial smoking bans with designated smoking rooms and ventilation systems do not protect people from the harm of SHS [3, 5, 6].

Although Japan is one of the first 40 parties that ratified the WHO FCTC, the progress on smoke-free regulations has been slow due to powerful opposition from the tobacco industry [7, 8]. Japan had no national smoke-free law prior to the revised Health Promotion Act, which was passed in July 2018 and took effect in stages by April 1, 2020 [9]. The new law is, however, less than comprehensive; all forms of smoking are prohibited in certain public places such as government offices, healthcare facilities, and schools, but outdoor smoking areas are permitted on the premises. Indoor smoking is also prohibited in other public places such as workplaces, restaurants, cafes, bars, and pachinko parlors (slot casinos), but designated smoking rooms are allowed. Furthermore, the law does not apply to private places such as homes and vehicles, rendering smoke-free rules in these places voluntary efforts with no penalties for failing to restrict SHS.

The Health Japan 21 (second term), a national health promotion program to prevent premature death and extend healthy life expectancy, set the reduction of SHS exposure as one of the 53 objectives to be tracked between fiscal years 2013 and 2022 (later extended to 2023) [10, 11]. Initially, the objective was set to achieve the target prevalence of monthly SHS exposure by 2022 at healthcare facilities (0%), government offices (0%), and restaurants, cafes, and bars combined (15%): and daily SHS exposure in the homes (3%). The target was separately specified for workplaces as to “Eliminate SHS” (i.e., 0%) by 2020. An interim evaluation conducted in 2018 revealed that these targets were unlikely to be achieved by 2020 or 2022; SHS exposure was on the decline but remained prevalent in all five places assessed (healthcare facilities, 5.4%; government offices, 7.0%; homes, 6.4%; restaurants/cafes/bars, 36.9%; and workplaces, 28.0%) [12]. In response to these results and the enforcement of the revised Health Promotion Act, the place-specific targets were removed with the intent of having a single objective to “Eliminate unwanted exposure to SHS in society” by 2022. However, the idea that there is “wanted” or “unwanted” SHS exposure has posed a severe public health threat by placing the responsibility for protecting people from the harms of SHS on individual choice and allowing the tobacco industry to justify public smoking.

Furthermore, the ambiguity of the term “unwanted exposure” complicates defining, monitoring, and evaluating the progress toward a smoke-free society. Prevalence of SHS exposure was monitored by the National Health and Nutrition Survey [10, 11], a nationally representative survey, with the question “*In the past month, have you had opportunities to inhale smoke from tobacco that was smoked by people other than yourself?*” before 2018; since 2018 and onward, the question has been modified to specifically ask about “*smoke from tobacco that was unwanted and smoked by people other than yourself*” in line with the revised national target. However, there is concern that this double-barreled question may reduce the validity and reliability of the assessment of SHS exposure for monitoring progress toward a smoke-free society. In this study, we examined SHS exposure that occurred despite the efforts to avoid it (i.e. unavoidable SHS exposure) as a proxy for “unwanted SHS exposure”. The prevalence of unavoidable SHS exposure observed in this study was compared to the national target for reducing SHS exposure – which should be 0% if unwanted SHS exposure is eliminated. As the second term of Health Japan 21 is ending, timely surveillance data is critical to inform tobacco control programs in the next decade aimed at protecting the public from the harm of SHS. The objectives of this study were 1) to estimate the prevalence and correlates of unavoidable exposure to SHS in indoor places and 2) to identify major places where unavoidable SHS exposure occurs.

## Methods

### Data source

We obtained data from the 2022 wave of the Japan Society and New Tobacco Internet Survey (JASTIS), a nationwide, Internet-based, self-reported survey of individuals aged 15 or older in Japan. Respondents were drawn from a pool of 2.3 million individuals of a private vendor, Rakuten Insight Inc. [13], by considering wide-ranging demographic and socioeconomic variables including education, housing, and marital status as defined by the Japan census [14]. Online informed consent was obtained from each respondent at the time of study registration. A more in-depth methodology has been reported elsewhere [14]. The latest 2022 data collection started on February 1 and closed on February 28, 2022, when the target sample size of 33,000 individuals was reached. The sample size was pre-determined to obtain a sufficient number of valid responses for stratified analysis by major demographic and socioeconomic characteristics [14].

### Measures

#### Exposure to secondhand smoke

Respondents were asked “*In the past month, have you had opportunities to inhale smoke from tobacco (excluding heated tobacco products) that was smoked by people other than yourself? Choose one that applies for each of the following places.*” This was separately asked for the following eight indoor places: home, car, restaurant, cafe, bar, workplace, school, and pachinko parlor (slot casino). The response categories included “*daily*”, “s*everal times in a week*”, “*about once in a week*”, “*about once in a month*”, “*less frequent or none*”, and “*I did not go to this place*”. Prevalence of SHS exposure was examined for different frequencies: daily and ≥monthly (reported “*about once in a month*” or more). Those who reported “*I did not go to this place*” were excluded from the denominator when examining the prevalence for the respective places. SHS exposures at restaurants, cafes, and bars were assessed separately and collectively, given that the national target for reducing SHS exposure is set for these three places combined [9]. An aggregate variable was also created to assess SHS exposure at any (≥1) of the eight indoor places. In the Results and Discussion sections, estimates will be presented for any indoor place unless otherwise specified.

#### Unavoidable exposure to secondhand smoke

Respondents were asked “*In the past month, have you practiced the following behavior? – Avoided tobacco smoke (secondhand smoke).*” The response categories included “*always*”, “*sometimes*”, “*scarcely*”, and “*never*”. Unavoidable SHS exposure was defined as exposure to SHS despite the respondent’s effort to avoid SHS “*always*” in the past month.

#### Sociodemographic and behavioral variables

Age, sex, education (less than high school/high school [including current students]/some college, college or higher), employment status (full-time/self-employed/part-time/unemployed), and the number of household members (including self) were assessed as possible correlates of unavoidable SHS exposure. Respondents were classified into current (past-30-day), former, or never smokers of any combustible tobacco products such as cigarettes, cigars, little cigars, pipes, and water pipes. Current use of heated tobacco products (HTPs) such as IQOS, Ploom Tech, Ploom S, Ploom X, glo, and lil, and alcohol drinking were also assessed.

### Statistical analysis

This study was a cross-sectional analysis. Individuals who provided unreasonable answers (N = 2,870) were excluded from the analysis by using a set of questions incorporated in the questionnaire [14]. For example, individuals who checked all multiple-choice items for illegal substance use (7 items) or presence of chronic conditions (15 items), those who answered with the same number over an entire set of questions, or those who chose a wrong answer for the question “*Choose the second item from the bottom*” were excluded. We also excluded respondents aged 75+ years (N = 2,006). The analysis was restricted to 25,672 individuals who had visited at least one of the eight indoor places in the past month.

The following three populations (with overlap) were used as the denominator: overall respondents (N = 25,672), those who always tried to avoid SHS (N = 14,971), and never smokers who always tried to avoid SHS (N = 10,416). We estimated the prevalence of SHS exposure by population characteristics and by place, and the latter was compared to the place-specific target prevalence determined by Health Japan 21 (second term). Among those who reported SHS exposure at any (≥1) of the eight indoor places, we explored the major sources of unavoidable exposure by computing the percentage of respondents who reported SHS exposure for the respective places. Multivariable Poisson regression was used to examine factors associated with unavoidable SHS exposure. Adjusted prevalence ratios (APRs) and 95% confidence intervals (CIs) were estimated by controlling for the aforementioned sociodemographic and behavioral variables.

All analyses were weighted to account for the selectivity of the Internet-based sample by applying inverse probability weighting (IPW) [14, 15]. To obtain IPW, propensity scores for “being an Internet survey respondent” was calculated by fitting logistic regression models adjusted for basic demographic, socioeconomic, health-related, and tobacco-use-related factors between the 2022 JASTIS sample and a nationally representative sample [16]. Further details regarding weighting have been reported elsewhere [14, 15]. All statistical analyses were performed using R version 4.1.3.

## Results

The characteristics of the respondents overall (N = 25,672), those who always tried to avoid SHS (N = 14,971), and never smokers who always tried to avoid SHS (N = 10,416) are presented in Supplementary Table 1. 56.9% of all respondents reported they always tried to avoid SHS in the past month; 13.9%, 11.4%, and 17.8% reported they avoided SHS sometimes, scarcely, and never, respectively.

12.4% of all respondents reported daily exposure to SHS (Table 1). The prevalence of daily unavoidable SHS exposure was 5.7% among those who always tried to avoid SHS and 4.2% among never smokers who always tried to avoid SHS. Overall, the likelihood of daily SHS exposure was significantly lower among those who avoided SHS always (APR = 0.38 [95%CI = 0.32–0.45]), sometimes (APR = 0.69 [95%CI = 0.59–0.80]), or scarcely (APR = 0.78 [95%CI = 0.68–0.89]) compared to those who never avoided SHS. Similarly, ≥monthly SHS exposure was reported by 34.0%, 21.4%, and 17.5% of overall, those who always tried to avoid SHS, and never smokers who always tried to avoid SHS, respectively (Supplementary Table 2). The likelihood of ≥monthly SHS exposure was significantly lower among those who always tried to avoid SHS (APR = 0.64 [95%CI = 0.59–0.69]) than those who never avoided SHS.

**Table 1:** Prevalence and adjusted prevalence ratios of daily exposure to secondhand smoke, 2022, Japan

	**Daily SHS exposure**					

	**Overall (N = 25,672)**		**Those who always tried ** **to avoid SHS (N = 14,971)**		**Never smokers who always tried ** **to avoid SHS (N = 10,416)**	

	**% (SE)**	**APR (95% CI)**	**% (SE)**	**APR (95% CI)**	**% (SE)**	**APR (95% CI)**
Overall	12.4 (0.4)	-	5.7 (0.4)	-	4.2 (0.4)	-
Age, years						
16–19	10.8 (1.9)	**2.19 (1.48–3.23)**	10.3 (2.4)	**3.09 (1.71–5.59)**	10.8 (2.5)	**4.97 (2.53–9.75)**
20–29	13.1 (0.7)	**1.39 (1.12–1.74)**	7.3 (0.7)	**1.71 (1.11–2.63)**	4.2 (0.5)	**2.08 (1.20–3.60)**
30–39	15.8 (1.1)	**1.49 (1.19–1.86)**	6.9 (0.9)	**1.59 (1.02–2.48)**	4.2 (0.8)	**2.09 (1.17–3.72)**
40–49	14.3 (0.8)	**1.30 (1.05–1.60)**	5.4 (0.6)	1.26 (0.84–1.90)	3.5 (0.6)	**1.97 (1.09–3.55)**
50–59	14.4 (1.0)	**1.37 (1.11–1.70)**	5.4 (1.0)	1.31 (0.85–2.03)	4.2 (0.9)	**2.36 (1.31–4.25)**
60–74	7.2 (0.7)	Ref.	3.6 (0.8)	Ref.	1.6 (0.4)	Ref.
Sex						
Female	10.7 (0.5)	Ref.	5.6 (0.5)	Ref.	4.0 (0.4)	Ref.
Male	14.1 (0.6)	**1.58 (1.38–1.80)**	5.9 (0.7)	**1.51 (1.14–2.01)**	4.5 (0.8)	1.05 (0.70–1.58)
Education						
Less than high school	22.1 (3.0)	**2.27 (1.76–2.93)**	15.4 (4.4)	**3.27 (2.02–5.31)**	9.7 (4.4)	**2.37 (1.03–5.44)**
High school	14.3 (0.6)	**1.30 (1.17–1.44)**	4.9 (0.5)	0.99 (0.76–1.31)	4.3 (0.6)	1.04 (0.69–1.56)
Some college/college or higher	10.2 (0.4)	Ref.	5.4 (0.4)	Ref.	4.1 (0.4)	Ref.
Employment status						
Full time	17.0 (0.7)	**1.62 (1.34–1.96)**	7.2 (0.8)	**1.61 (1.08–2.40)**	4.3 (0.6)	1.25 (0.75–2.09)
Self-employed	13.4 (1.9)	1.24 (0.93–1.65)	6.2 (2.5)	1.30 (0.78–2.16)	1.9 (0.8)	0.73 (0.30–1.81)
Part time	11.4 (0.7)	**1.26 (1.04–1.54)**	6.5 (0.9)	1.48 (0.99–2.20)	5.5 (0.9)	1.35 (0.86–2.12)
Unemployed	7.3 (0.6)	Ref.	3.8 (0.5)	Ref.	3.4 (0.6)	Ref.
Number of household members						
1	11.6 (0.9)	Ref.	6.3 (1.3)	Ref.	3.8 (0.9)	Ref.
2	10.7 (0.7)	**1.25 (1.04–1.51)**	4.1 (0.6)	1.08 (0.69–1.69)	2.7 (0.6)	1.13 (0.60–2.12)
3+	13.6 (0.5)	**1.25 (1.07–1.46)**	6.6 (0.6)	1.17 (0.83–1.65)	5.1 (0.6)	1.27 (0.79–2.04)
Smoking status						
Never	5.8 (0.4)	Ref.	4.2 (0.4)	Ref.	-	-
Former	12.8 (0.8)	**1.67 (1.41–1.99)**	6.4 (1.0)	**1.61 (1.18–2.20)**	-	-
Current	29.3 (1.2)	**2.56 (2.18–3.01)**	17.1 (2.5)	**2.12 (1.37–3.30)**	-	-
Current use of heated tobacco products						
No	8.8 (0.4)	Ref.	4.7 (0.4)	Ref.	4.0 (0.4)	Ref.
Yes	37.0 (1.5)	**2.12 (1.86–2.41)**	29.8 (3.8)	**4.22 (2.84–6.25)**	35.5 (9.9)	**8.27 (4.85–14.10)**
Alcohol use						
Non-current/never	11.4 (0.6)	Ref.	6.1 (0.6)	Ref.	4.9 (0.6)	Ref.
Current	13.5 (0.5)	0.99 (0.89–1.10)	5.3 (0.6)	0.87 (0.68–1.12)	3.1 (0.4)	0.88 (0.64–1.22)
Avoidance of SHS (past month)						
Always	5.7 (0.4)	**0.38 (0.32–0.45)**	-	-	-	-
Sometimes	14.9 (1.0)	**0.69 (0.59–0.80)**	-	-	-	-
Scarcely	20.8 (1.3)	**0.78 (0.68–0.89)**	-	-	-	-
Never	26.6 (1.3)	Ref.	-	-	-	-

**Abbreviations:** APR = adjusted prevalence ratio; CI = confidence interval; SE = standard error; SHS = secondhand smoke

**Note:** Data were derived from the Japan Society and New Tobacco Internet Survey (JASTIS) and weighted by applying inverse probability weighting to account for the selectivity of the Internet-based sample. Never smokers were individuals who had never smoked any combustible tobacco products (cigarettes, cigars, little cigars, pipes, and water pipes). Current smoking and product use were assessed as past-30-day smoking/use. Estimates in **bold** type indicate statistical significance.

Among those who always tried to avoid SHS, the likelihood of daily unavoidable SHS exposure was significantly higher in younger individuals with the highest APR among adolescents (aged 16–19) (APR = 3.09 [95%CI = 1.71–5.59] vs. age 60–74), males (APR = 1.51 [95%CI = 1.14–2.01], vs. females), those with less than high school education (APR = 3.27 [95%CI = 2.02–5.31], vs. ≥some college education), former and current smokers (APR = 1.61 [95%CI = 1.18–2.20] and APR = 2.12 [95%CI = 1.37–3.30], respectively, vs. never smokers), current HTP users (APR = 4.22 [95%CI = 2.84–6.25], vs. nonusers), and full-time workers (APR = 1.61 [95%CI = 1.08–2.40], vs. unemployed) (Table 1). When the analysis was limited to never smokers who always tried to avoid SHS, the likelihood of daily unavoidable SHS exposure remained significantly higher in several population subgroups. Specifically, never-smoking adolescents (aged 16–19) were 4.97 (95%CI = 2.53–9.75) times more likely to report daily unavoidable exposure to SHS compared to those aged 60–74. Increased likelihoods were also observed in other age groups: APR = 2.08 (95%CI = 1.20–3.60), APR = 2.09 (95%CI = 1.17–3.72), APR = 1.97 (95%CI = 1.09–3.55), and APR = 2.36 (95%CI = 1.31–4.25) among those aged 20–29, 30–39, 40–49, and 50–59, respectively. Among never smokers, those with less than high school education had 2.37 (95%CI = 1.03–5.44) times increased likelihood of daily unavoidable SHS exposure than those with ≥some college education; the likelihood was 8.27 (95%CI = 4.85–14.10) times higher among current HTP users than nonusers. Among never smokers, age and HTP use were also significantly associated with ≥monthly unavoidable exposure to SHS, but education was not; increased likelihoods were also seen among full-time (APR = 1.62 [95%CI = 1.32–1.99]) and part-time workers (APR = 1.67 [95%CI = 1.36–2.05]) compared to unemployed individuals (Supplementary Table 2).

**Fig. 1:**
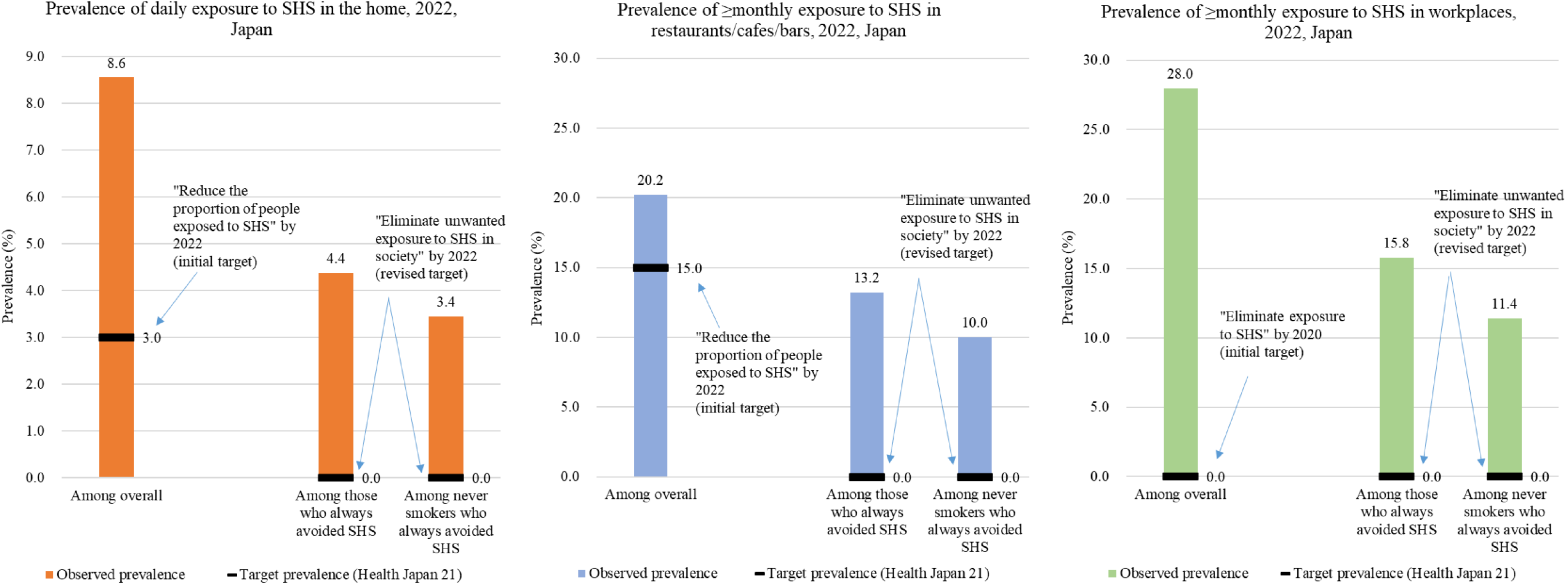
Comparison of the observed and national target prevalence of exposure to secondhand smoke, 2022, Japan **Abbreviations:** SHS = secondhand smoke **Note:** Data were derived from the Japan Society and New Tobacco Internet Survey (JASTIS) and weighted by applying inverse probability weighting to account for the selectivity of the Internet-based sample. The national target prevalence is that of Health Japan 21 (second term). Never smokers were individuals who had never smoked any combustible tobacco products (cigarettes, cigars, little cigars, pipes, and water pipes).

Figure 1 compares the observed and target prevalence of SHS exposure in the home, restaurants/cafes/bars, and workplaces among those who visited the respective places in the past month. In the home, the overall prevalence of daily SHS exposure was 8.6%, exceeding the place-specific target of 3.0% initially set by Health Japan 21 (second term). Daily unavoidable SHS exposure in the home was reported by 4.4% and 3.4% of respondents who always tried to avoid SHS and never smokers who always tried to avoid SHS, respectively; neither of which reached the revised target of “*Eliminate unwanted exposure to SHS in society by 2022*” (= 0%). Similarly, the overall prevalence of ≥monthly SHS exposure in restaurants/cafes/bars was 20.2%. ≥Monthly unavoidable SHS exposure was reported by 13.2% and 10.0% of respondents who always tried to avoid SHS and never smokers who always tried to avoid SHS, respectively. All of these estimates exceeded the target prevalence set by Health Japan 21 (second term). In workplaces, the prevalence of ≥monthly SHS exposure was 28.0% among overall. ≥Monthly unavoidable SHS exposure was reported by 15.8% and 11.4% of respondents who always tried to avoid SHS and never smokers who always tried to avoid SHS, respectively. All of these estimates exceeded the national target. The prevalence of SHS exposure in all eight indoor places is shown in Table 2. Among never smokers, the highest prevalence of daily unavoidable SHS exposure was seen in the home (3.4%), workplaces (2.3%), and pachinko parlors (1.3%); for ≥monthly unavoidable SHS exposure, the highest prevalence was seen in workplaces (11.4%), restaurants/cafes/bars (10.0%), and the home (7.6%).

**Table 2:** Prevalence of exposure to secondhand smoke by place, 2022, Japan

	**Denominator**			**Prevalence – Daily SHS exposure**			**Prevalence – ≥Monthly ** **SHS exposure**		

	**Overall**	**Those who always ** **tried to ** **avoid SHS**	**Never smokers ** **who always tried to ** **avoid SHS**	**Overall**	**Those who always ** **tried to ** **avoid SHS**	**Never ** **smokers ** **who always ** **tried to ** **avoid SHS**	**Overall**	**Those who always ** **tried to ** **avoid SHS**	**Never smokers ** **who always tried to ** **avoid SHS**
	**N**	**N**	**N**	**% (SE)**	**% (SE)**	**% (SE)**	**% (SE)**	**% (SE)**	**% (SE)**
Any indoor place – Total	25672	14971	10416	12.4 (0.4)	5.7 (0.4)	4.2 (0.4)	34.0 (0.6)	21.4 (0.6)	17.5 (0.7)
Home	23193	13446	9358	8.6 (0.4)	4.4 (0.4)	3.4 (0.4)	16.8 (0.4)	9.8 (0.5)	7.6 (0.5)
Car	20788	12107	8245	2.6 (0.2)	1.0 (0.2)	0.6 (0.2)	13.0 (0.4)	6.2 (0.4)	4.2 (0.4)
Restaurant/cafe/bar combined	16640	9208	6428	1.0 (0.1)	0.5 (0.1)	0.3 (0.2)	20.2 (0.6)	13.2 (0.7)	10.0 (0.6)
Restaurant	15356	8590	6020	0.4 (0.1)	0.4 (0.1)	0.3 (0.2)	13.2 (0.5)	8.6 (0.6)	6.5 (0.5)
Cafe	11853	6438	4579	0.8 (0.1)	0.5 (0.2)	0.4 (0.2)	16.4 (0.6)	10.5 (0.7)	7.6 (0.7)
Bar	8504	4060	2724	1.2 (0.2)	0.7 (0.3)	0.6 (0.4)	28.6 (0.9)	20.0 (1.1)	14.5 (1.1)
Workplace	17881	9632	6675	9.4 (0.4)	3.8 (0.4)	2.3 (0.3)	28.0 (0.6)	15.8 (0.7)	11.4 (0.6)
School	7801	4386	3293	1.1 (0.2)	1.0 (0.3)	0.8 (0.4)	12.2 (0.6)	7.0 (0.7)	4.7 (0.6)
Pachinko parlor (slot casino)	5164	2360	1509	2.2 (0.4)	1.7 (0.5)	1.3 (0.7)	26.9 (1.1)	14.7 (1.5)	7.0 (1.0)

**Abbreviations:** SE = standard error; SHS = secondhand smoke

**Note:** Data were derived from the Japan Society and New Tobacco Internet Survey (JASTIS) and weighted by applying inverse probability weighting to account for the selectivity of the Internet-based sample. The denominator was individuals who had visited the respective places in the past 30 days. Never smokers were individuals who had never smoked any combustible tobacco products (cigarettes, cigars, little cigars, pipes, and water pipes).

**Fig. 2:**
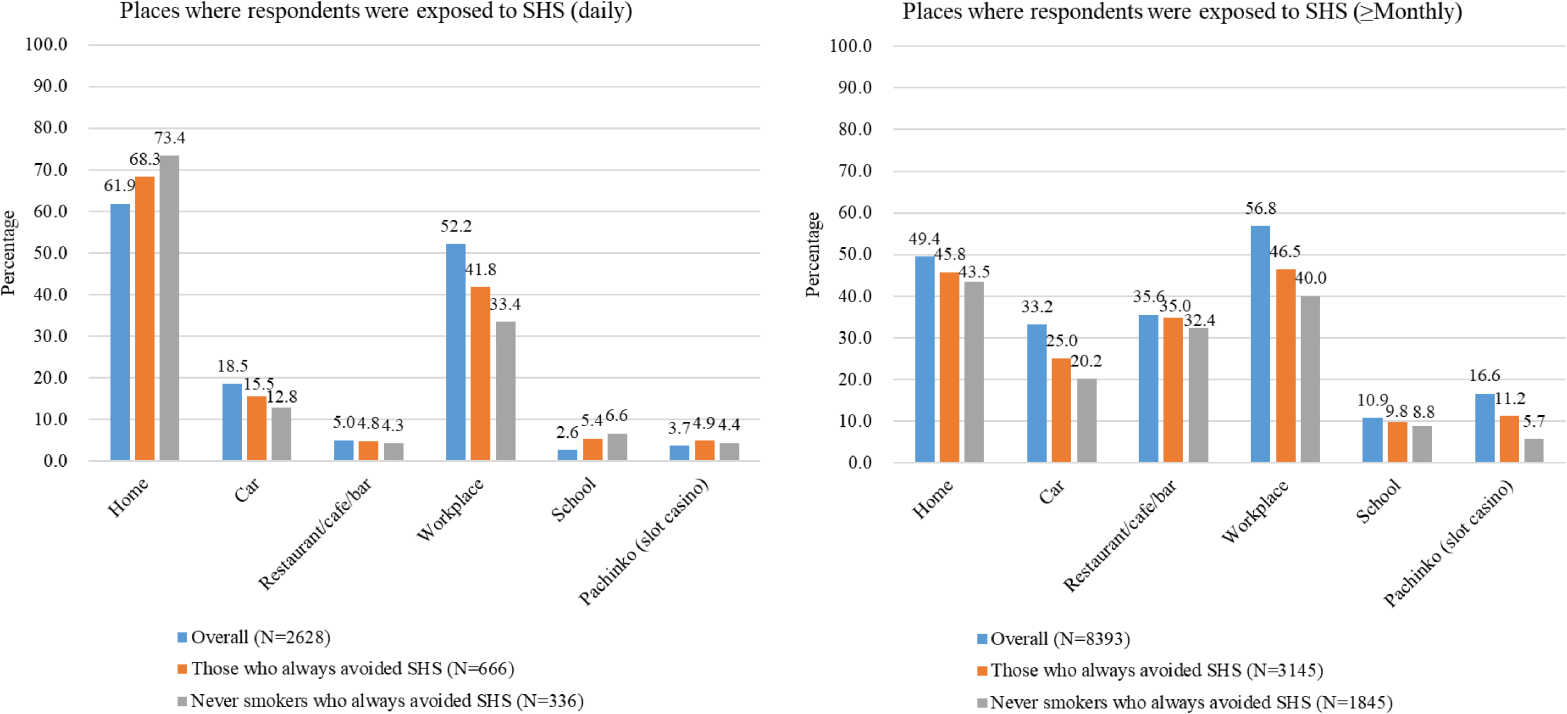
Percentage of respondents who were exposed to secondhand smoke in specific indoor places, 2022, Japan **Abbreviations:** SHS = secondhand smoke **Note:** Data were derived from the Japan Society and New Tobacco Internet Survey (JASTIS) and weighted by applying inverse probability weighting to account for the selectivity of the Internet-based sample. The denominator was individuals who were exposed to secondhand smoke in any (≥1) indoor places in the past month. Never smokers were individuals who had never smoked any combustible tobacco products (cigarettes, cigars, little cigars, pipes, and water pipes).

Figure 2 shows the percentage of respondents who were exposed to SHS in specific indoor places among those who were exposed to SHS in any (≥1) indoor places in the past month. The estimates are hereinafter presented in the following order: of all exposed respondents, exposed respondents who always tried to avoid SHS, and exposed respondents who had never smoked and always tried to avoid SHS. For daily SHS exposure, the most common place was home (61.9%, 68.3%, and 73.4%, respectively), with the highest percentage among never smokers. Other common places included workplace (52.2%, 41.8%, and 33.4%, respectively) and car (18.5%, 15.5%, and 12.8%, respectively), with the lowest percentages among never smokers. For ≥monthly SHS exposure, common places included workplace (56.8%, 46.5%, and 40.0%, respectively), home (49.4%, 45.8%, and 43.5%, respectively), and restaurant/cafe/bar (35.6%, 35.0%, and 32.4%, respectively) with the lowest percentages among never smokers.

## Discussion

In 2022, although the majority of respondents (56.9%) always tried to avoid SHS in the past month, 5.7% of them reported daily exposure to SHS, and 21.4% reported being exposed to SHS at least once a month. Even among never smokers who always tried to avoid SHS, the prevalence of unavoidable SHS exposure was 4.2% (daily) and 17.5% (at least once a month). These results suggest that the lack of comprehensive smoke-free laws in Japan provides inadequate protection against SHS that cannot be complemented by individual efforts. The authorities must not leave the responsibility for “running away” from SHS to the individual level, but must implement comprehensive smoke-free regulations to protect the public from the harms of SHS.

We found that daily unavoidable SHS exposure was disproportionately high among younger individuals including adolescents. SHS contains neurotoxic substances such as lead, arsenic, and several other heavy metals that can impair cognitive development and function in children and youth [3]. Several anecdotal studies also suggest an association between SHS exposure and academic failure among non-smoking adolescents [17–20]. Combined with the finding that those with lower levels of education were also prone to have unavoidable SHS exposure, these demographic and socioeconomic variations may contribute to health disparities. It is of utmost importance to comply with WHO FCTC and ensure a 100% smoke-free environment for all. According to the results from the 2018 International Tobacco Control (ITC) Japan Survey, the majority of Japanese respondents (65–81%) were in favor of a complete smoking ban in indoor public places such as workplaces, restaurants, and bars before the implementation of the revised Health Promotion Act [21]. Given the global tendency that public support for comprehensive smoke-free laws increases after they are implemented in a country [22–24], public support is unlikely a barrier to smoke-free action in Japan. It is also important to promote smoke-free environment in private spaces such as homes and vehicles to protect people from SHS. One of the principles of the 2020 revision of the Health Promotion Act was to protect vulnerable populations, such as minors and people in poor health, from the harm of SHS by implementing stricter smoke-free regulations. Although the revised law bans smoking in indoor public places such as schools and healthcare facilities, there are currently no smoking restrictions in private spaces, where vulnerable people are likely to spend much of their time. A better understanding of the sources of SHS exposure for the vulnerable is warranted to inform future interventions.

We also found a strong association between HTP use and unavoidable exposure to SHS. Japan is a major market for HTP, which is currently the second most-used tobacco product after conventional cigarettes with a national prevalence of 11.8% [25]. HTPs are subject to WHO FCTC measures [26], and the new smoke-free regulations of the revised Health Promotion Act have been extended to include HTPs [9]. Since 2018, some public facilities have set up designated rooms exclusively for the use of HTPs and vapor products, where eating and drinking are also allowed, as a transitional measure to comply with the law [27]. However, combustible tobacco products and HTPs are not separated in many indoor facilities that equip “smoking” rooms where the use of any tobacco or tobacco-like products is permitted [27]. HTP users may have increased opportunities to be exposed to SHS unavoidably by utilizing these designated rooms. A detailed understanding of the behavioral patterns of HTP users is essential to inform smoke-free policies and make recommendations on whether HTPs should be subject to the same regulations as combustible tobacco products.

By place, home and workplace were the dominant sources of unavoidable SHS exposure, as seen in the location profile reported by those who experienced unavoidable SHS exposure. The extent of SHS exposure in these places may be magnified by the relatively confined nature of private residences and office buildings and the longer durations of exposure, given that most workers, children, and youth have limited control over their own exposure to SHS [3, 28]. Although not addressed in Health Japan 21, unavoidable SHS exposures in cars and pachinko parlors are not negligible. In particular, the levels of SHS contaminants can be extremely high in enclosed environments such as vehicles [29–31]. We found that car was the third most common source of daily unavoidable SHS exposure after home and workplace, and the fourth of ≥monthly unavoidable SHS exposure, with more than 20% of never smokers unavoidably exposed to SHS reporting that the exposure occurred in cars. Furthermore, comparisons of the prevalence of SHS exposure observed in this study to the national target prevalence for home, restaurant/cafe/bar, and workplace revealed that none of these targets were achieved by 2022. These findings underscore the importance of implementing smoke-free home and vehicle rules in conjunction with the enhanced implementation of comprehensive smoke-free laws in public places.

To our best knowledge, there are no countries other than Japan that specifically aim at reducing “unwanted” SHS exposure, as exposure to known harms is not a choice made at the individual level, but to be regulated by the authorities. In this study, unavoidable SHS exposure was examined as a proxy for “unwanted SHS exposure”, but these two concepts are not entirely interchangeable. SHS-avoiding behavior (the “action” stage of the transtheoretical model) [32] and its continuation (always avoiding SHS, the “maintenance” stage) are the results of one’s knowledge about the harms of SHS (the “pre-contemplation” or “contemplation” stage), risk assessment (the “contemplation” stage), and/or decision to avoid SHS (the “preparation” stage), while unwanted SHS exposure can occur to people at any of these stages. Thus, unavoidable SHS exposure in this study can be interpreted as a narrowly defined measure of unwanted SHS exposure. However, since it is unlikely for people to actively expose themselves to SHS and that the evidence is clear about the harms of SHS, national tobacco control must ensure a smoke-free environment for all, regardless of whether people voluntary take actions to avoid SHS.

This study has several limitations. First, because the sample was collected through Internet-based recruitment, our findings may not be generalizable to populations with limited Internet access or literacy. However, over 90% of the Japanese population had access to the Internet as of 2021 [33], and this study used weighted data to address differences in key socioeconomic and demographic characteristics and tobacco use behavior between the respondents of this Internet survey and a nationally representative population. Although we are unable to assess the extent of residual selection bias that could not be addressed by post-hoc weighting, this study provides an overview of the SHS exposure profile and highlights vulnerability in specific populations. Second, since unavoidable SHS exposure in this study was assessed as a consequence of the behavior to avoid SHS rather than attitudes toward SHS, it may not entirely correspond to “unwanted SHS exposure” which is of interest to the national health promotion program. Third, this study assessed eight indoor places but did not cover all types of indoor venues for which national targets were initially set by Health Japan 21, such as government buildings and healthcare facilities. However, providing data on SHS exposure in cars and pachinko parlors is a strength because data for these places are scarce. Lastly, the self-reported nature of the survey might have led to recall bias and misunderstanding of the questions (e.g. confusing SHS with secondhand emissions from non-combustible tobacco or tobacco-like products) although the questionnaire was designed to minimize such inaccuracy.

## Conclusions

In summary, in 2022, nearly 6 in 10 respondents always tried to avoid SHS, with 5.7% of them unavoidably exposed to SHS daily and 21.4% at least once a month. The prevalence of unavoidable SHS exposure was not zero even among never smokers who always tried to avoid SHS (4.2% exposed daily and 17.5% at least once a month). The prevalence of unavoidable SHS exposure in the home, restaurants/cafes/bars, and workplaces did not achieve the targets of Health Japan 21 (second term). Implementing smoke-free home and vehicle rules in conjunction with the enforcement of comprehensive smoke-free laws in public places is essential to protect the public from the harm of SHS.

## Abbreviations

APR: Adjusted prevalence ratio
CI: Confidence interval
FCTC: Framework Convention on Tobacco Control
HTP: Heated tobacco product
IPW: Inverse probability weight
JASTIS: Japan Society and New Tobacco Internet Survey
SHS: Secondhand smoke
WHO: World Health Organization

## Supplementary information

**Additional file 1:** **Supplementary Table 1.** Characteristics of respondents, 2022, Japan. **Supplementary Table 2.** Prevalence and adjusted prevalence ratios of ≥monthly exposure to secondhand smoke, 2022, Japan.

## Declarations

### Availability of data and material

The data are not publicly available due to privacy restrictions and only available on request to the corresponding author at tabuchitak@gmail.com.
