# Supplementary material for: Unavoidable exposure to secondhand smoke in indoor places: a cross-sectional comparison to the Health Japan 21 (second term) objectives, 2022
Source: Environ Health Prev Med. 2023 Jul 12;28:45. doi: 10.1265/ehpm.23-00055 (PMC10366352; doi:10.1265/ehpm.23-00055)
Supplement: Supplementary file 1 — Additional file 1: Supplementary Table 1. Characteristics of respondents, 2022, Japan. Supplementary Table 2. Prevalence and adjusted prevalence ratios of ≥monthly exposure to secondhand smoke, 2022, Japan. [file ehpm-28-045-s001.docx]

**Supplementary Table 1.** Characteristics of respondents, 2022, Japan

|  | Overall | | |  | | Those who always avoided SHS | | | |  | | Never smokers who always avoided SHS | | | |
| --- | --- | --- | --- | --- | --- | --- | --- | --- | --- | --- | --- | --- | --- | --- | --- |
|  | Distribution  N | Distribution  % | Percentage that reported "Always" avoiding SHS  % (SE) | | Distribution  N | | Distribution  % | |  | | Distribution  N | | Distribution  % | |  |
| Overall | 25672 | 100.0 | 56.9 (0.6) |  | | 14971 | | 100.0 | |  | | 10416 | | 100.0 | |
| Age, years |  |  |  |  | |  | |  | |  | |  | |  | |
| 16-19 | 646 | 6.9 | 64.8 (3.2) |  | | 434 | | 7.9 | |  | | 417 | | 11.0 | |
| 20-29 | 5491 | 12.2 | 50.9 (1.0) |  | | 2912 | | 10.9 | |  | | 2496 | | 13.1 | |
| 30-39 | 3726 | 15.7 | 51.7 (1.5) |  | | 2100 | | 14.3 | |  | | 1561 | | 14.8 | |
| 40-49 | 4867 | 21.1 | 52.7 (1.1) |  | | 2675 | | 19.6 | |  | | 1761 | | 18.8 | |
| 50-59 | 4255 | 19.4 | 55.0 (1.3) |  | | 2353 | | 18.8 | |  | | 1524 | | 17.5 | |
| 60-74 | 6687 | 24.7 | 66.0 (1.2) |  | | 4497 | | 28.6 | |  | | 2657 | | 24.7 | |
| Sex |  |  |  |  | |  | |  | |  | |  | |  | |
| Female | 13013 | 50.4 | 67.2 (0.8) |  | | 8727 | | 59.5 | |  | | 7072 | | 69.9 | |
| Male | 12659 | 49.6 | 46.5 (0.8) |  | | 6244 | | 40.5 | |  | | 334 | | 30.1 | |
| Education |  |  |  |  | |  | |  | |  | |  | |  | |
| Less than high school | 500 | 6.3 | 52.6 (3.6) |  | | 216 | | 5.8 | |  | | 125 | | 4.2 | |
| High school | 6684 | 42.2 | 55.0 (0.9) |  | | 3741 | | 40.9 | |  | | 2526 | | 40.0 | |
| Some college/college or higher | 18355 | 51.5 | 58.9 (0.5) |  | | 10949 | | 53.3 | |  | | 7715 | | 55.8 | |
| Employment status |  |  |  |  | |  | |  | |  | |  | |  | |
| Full time | 10981 | 38.3 | 47.5 (0.8) |  | | 5614 | | 32.0 | |  | | 3742 | | 28.6 | |
| Self-employed | 1633 | 6.9 | 47.0 (2.5) |  | | 820 | | 5.7 | |  | | 432 | | 3.9 | |
| Part time | 5811 | 24.2 | 59.8 (1.3) |  | | 3511 | | 25.4 | |  | | 2661 | | 28.3 | |
| Unemployed | 7247 | 30.7 | 68.5 (1.1) |  | | 5026 | | 37.0 | |  | | 3581 | | 39.2 | |
| Number of household members |  |  |  |  | |  | |  | |  | |  | |  | |
| 1 | 5713 | 16.1 | 51.1 (1.5) |  | | 3005 | | 14.4 | |  | | 2149 | | 14.0 | |
| 2 | 7743 | 30.6 | 61.2 (1.1) |  | | 4818 | | 33.0 | |  | | 3164 | | 31.7 | |
| 3+ | 12216 | 53.3 | 56.2 (0.7) |  | | 7148 | | 52.6 | |  | | 5103 | | 54.3 | |
| Smoking status |  |  |  |  | |  | |  | |  | |  | |  | |
| Never | 14617 | 54.2 | 71.6 (0.7) |  | | 10416 | | 68.3 | |  | | - | |  | |
| Former | 6333 | 25.4 | 54.0 (1.1) |  | | 3516 | | 24.1 | |  | | - | |  | |
| Current | 4722 | 20.4 | 21.3 (1.1) |  | | 1039 | | 7.6 | |  | | - | |  | |
| Current use of heated tobacco products |  |  |  |  | |  | |  | |  | |  | |  | |
| No | 22652 | 87.3 | 62.5 (0.6) |  | | 14403 | | 96.0 | |  | | 10338 | | 99.3 | |
| Yes | 3020 | 12.7 | 18.1 (1.3) |  | | 568 | | 4.0 | |  | | 78 | | 0.7 | |
| Alcohol use |  |  |  |  | |  | |  | |  | |  | |  | |
| Non-current/never | 12163 | 50.5 | 60.5 (0.8) |  | | 7573 | | 53.7 | |  | | 5910 | | 60.8 | |
| Current | 13509 | 49.5 | 53.2 (0.8) |  | | 7398 | | 46.3 | |  | | 4506 | | 39.2 | |
| Avoidance of SHS (past month) |  |  |  |  | |  | |  | |  | |  | |  | |
| Always | 14971 | 56.9 | - |  | | - | | - | |  | | - | | - | |
| Sometimes | 3853 | 13.9 | - |  | | - | | - | |  | | - | | - | |
| Scarcely | 2771 | 11.4 | - |  | | - | | - | |  | | - | | - | |
| Never | 4077 | 17.8 | - |  | | - | | - | |  | | - | | - | |

**Abbreviations:** SE=standard error; SHS=secondhand smoke

**Note:** Data were derived from the Japan Society and New Tobacco Internet Survey (JASTIS) and weighted by applying inverse probability weighting to account for the selectivity of the Internet-based sample. Never smokers were individuals who had never smoked any combustible tobacco products (cigarettes, cigars, little cigars, pipes, and water pipes). Current smoking and product use were assessed as past-30-day smoking/use.

**Supplementary Table 2.** Prevalence and adjusted prevalence ratios of ≥monthly exposure to secondhand smoke, 2022, Japan

|  | ≥Monthly SHS exposure | | | | | | | |
| --- | --- | --- | --- | --- | --- | --- | --- | --- |
|  | Overall (N=25,672) | |  | Those who always avoided SHS (N=14,971) | |  | Never smokers who always avoided SHS (N=10,416) | |
|  | % (SE) | APR (95% CI) |  | % (SE) | APR (95% CI) |  | % (SE) | APR (95% CI) |
| Overall | 34.0 (0.6) | - |  | 21.4 (0.6) | - |  | 17.5 (0.7) | - |
| Age, years |  |  |  |  |  |  |  |  |
| 16-19 | 32.7 (2.9) | **1.81 (1.51-2.18)** |  | 27.6 (3.3) | **2.01 (1.54-2.63)** |  | 25.1 (3.2) | **2.29 (1.65-3.19)** |
| 20-29 | 43.9 (1.0) | **1.57 (1.43-1.73)** |  | 31.6 (1.2) | **1.89 (1.61-2.22)** |  | 26.5 (1.2) | **2.17 (1.73-2.72)** |
| 30-39 | 41.7 (1.4) | **1.35 (1.22-1.50)** |  | 25.0 (1.5) | **1.42 (1.18-1.71)** |  | 19.3 (1.6) | **1.59 (1.23-2.06)** |
| 40-49 | 36.7 (1.1) | **1.22 (1.10-1.34)** |  | 22.2 (1.2) | **1.27 (1.07-1.51)** |  | 16.7 (1.3) | **1.48 (1.15-1.91)** |
| 50-59 | 34.1 (1.3) | **1.17 (1.05-1.29)** |  | 20.6 (1.6) | 1.2 (0.999-1.44) |  | 18.1 (1.9) | **1.53 (1.18-1.99)** |
| 60-74 | 22.3 (1.1) | Ref. |  | 14.0 (1.2) | Ref. |  | 8.5 (0.8) | Ref. |
| Sex |  |  |  |  |  |  |  |  |
| Female | 28.4 (0.7) | Ref. |  | 19.4 (0.8) | Ref. |  | 16.8 (0.8) | Ref. |
| Male | 39.8 (0.8) | **1.12 (1.05-1.19)** |  | 24.3 (1.0) | 1.08 (0.96-1.21) |  | 19.2 (1.2) | 1.03 (0.88-1.2) |
| Education |  |  |  |  |  |  |  |  |
| Less than high school | 41.7 (3.6) | **1.42 (1.22-1.65)** |  | 24.1 (4.9) | 1.33 (0.95-1.85) |  | 14.0 (4.7) | 1.01 (0.56-1.80) |
| High school | 36.0 (0.9) | **1.13 (1.07-1.19)** |  | 21.6 (1.0) | **1.12 (1.01-1.24)** |  | 17.7 (1.1) | 1.13 (0.97-1.31) |
| Some college/college or higher | 32.4 (0.5) | Ref. |  | 21.7 (0.6) | Ref. |  | 18.5 (0.7) | Ref. |
| Employment status |  |  |  |  |  |  |  |  |
| Full time | 44.1 (0.8) | **1.58 (1.44-1.74)** |  | 27.2 (1.0) | **1.67 (1.41-1.97)** |  | 22.0 (1.1) | **1.62 (1.32-1.99)** |
| Self-employed | 36.6 (2.4) | **1.40 (1.22-1.61)** |  | 24.0 (3.6) | **1.66 (1.26-2.18)** |  | 13.4 (2.6) | 1.12 (0.77-1.64) |
| Part time | 35.3 (1.2) | **1.49 (1.35-1.64)** |  | 25.8 (1.4) | **1.72 (1.46-2.02)** |  | 22.0 (1.4) | **1.67 (1.36-2.05)** |
| Unemployed | 19.8 (0.9) | Ref. |  | 13.0 (0.9) | Ref. |  | 11.5 (1.0) | Ref. |
| Number of household members |  |  |  |  |  |  |  |  |
| 1 | 36.5 (1.4) | Ref. |  | 23.3 (1.8) | Ref. |  | 19.9 (1.8) | Ref. |
| 2 | 28.5 (1.0) | 1.05 (0.97-1.14) |  | 16.4 (1.0) | 1.01 (0.85-1.20) |  | 12.4 (0.9) | 0.98 (0.78-1.23) |
| 3+ | 36.4 (0.7) | 1.05 (0.98-1.13) |  | 24.0 (0.8) | 1.11 (0.96-1.27) |  | 19.9 (1.0) | 1.07 (0.88-1.31) |
| Smoking status |  |  |  |  |  |  |  |  |
| Never | 21.8 (0.6) | Ref. |  | 17.5 (0.7) | Ref. |  | - | - |
| Former | 37.0 (1.1) | **1.45 (1.34-1.57)** |  | 25.8 (1.4) | **1.57 (1.38-1.77)** |  | - | - |
| Current | 62.8 (1.3) | **1.88 (1.74-2.02)** |  | 42.1 (2.9) | **1.69 (1.44-2.00)** |  | - | - |
| Current use of heated tobacco products |  |  |  |  |  |  |  |  |
| No | 28.1 (0.6) | Ref. |  | 19.6 (0.6) | Ref. |  | 17.2 (0.7) | Ref. |
| Yes | 75.0 (1.3) | **1.52 (1.44-1.61)** |  | 63.5 (4.0) | **2.08 (1.77-2.44)** |  | 59.4 (8.2) | **2.93 (2.16-3.95)** |
| Alcohol use |  |  |  |  |  |  |  |  |
| Non-current/never | 30.6 (0.8) | Ref. |  | 20.4 (0.9) | Ref. |  | 17.0 (0.8) | Ref. |
| Current | 37.5 (0.8) | **1.06 (1.01-1.11)** |  | 22.5 (0.9) | 1.05 (0.95-1.16) |  | 18.3 (1.1) | 1.13 (0.99-1.28) |
| Avoidance of SHS (past month) |  |  |  |  |  |  |  |  |
| Always | 21.4 (0.6) | **0.64 (0.59-0.69)** |  | - | - |  | - | - |
| Sometimes | 48.6 (1.5) | 1.07 (0.99-1.14) |  | - | - |  | - | - |
| Scarcely | 54.2 (1.7) | 1.04 (0.97-1.12) |  | - | - |  | - | - |
| Never | 50.1 (1.5) | Ref. |  | - | - |  | - | - |

**Abbreviations:** APR=adjusted prevalence ratio; CI=confidence interval; SE=standard error; SHS=secondhand smoke

**Note:** Data were derived from the Japan Society and New Tobacco Internet Survey (JASTIS) and weighted by applying inverse probability weighting to account for the selectivity of the Internet-based sample. Never smokers were individuals who had never smoked any combustible tobacco products (cigarettes, cigars, little cigars, pipes, and water pipes). Current smoking and product use were assessed as past-30-day smoking/use. Estimates in **bold** type indicate statistical significance.
